# Cognitive Interference Cancellation with Digital Channelizer for Satellite Communication

**DOI:** 10.3390/s20020355

**Published:** 2020-01-08

**Authors:** Byounghak Kim, Heejung Yu, Song Noh

**Affiliations:** 1 Radio and Satellite Research Division, Telecommunications and Media Research Laboratory, Electronics and Telecommunications Research Institute, Daejeon 34129, Korea; byounghak@etri.re.kr; 2 Department of Electronics and Information Engineering, Korea University, Sejong 30019, Korea; 3 Department of Information and Telecommunication Engineering, Incheon National University, Incheon 22012, Korea

**Keywords:** detection, interference cancellation, digital channelizer, satellite communication, cognitive communication

## Abstract

The concept of Internet of Things (IoT) has attracted much research attention for the realization of a smart society. However, the radio transmission coverage of the existing IoT solutions is not enough to connect lots of devices deployed over wide areas. Therefore, satellite networks have been considered as one of the most attractive solutions to wide cell coverage of IoT, i.e., global-scaled IoT. In satellite communication, a digital channelizer is one of the most significant parts that support multiple transponders. Owing to their wide coverage, satellite communication systems are more vulnerable to interference than other types of wireless communication systems. In this study, a cognitive interference cancellation using the inherent properties of a digital channelizer is considered. The proposed method detects a subchannel corrupted by interference and omits it. A simple energy detection method and a modified version are proposed for detection of interference. In the modified (i.e., improved) method, the number of required signal blocks to achieve the target detection performance can be reduced, i.e., the detection performance is improved with the same number of blocks, by exploiting the property of the fast Fourier transform (FFT) algorithm. Detection performance such as false alarm and detection probabilities are analyzed, and the validity of the analysis is verified with numerical results. It is also shown that an interference lower than a certain level in the proposed approach does not need to be cancelled.

## 1. Introduction

**Figure 1:**
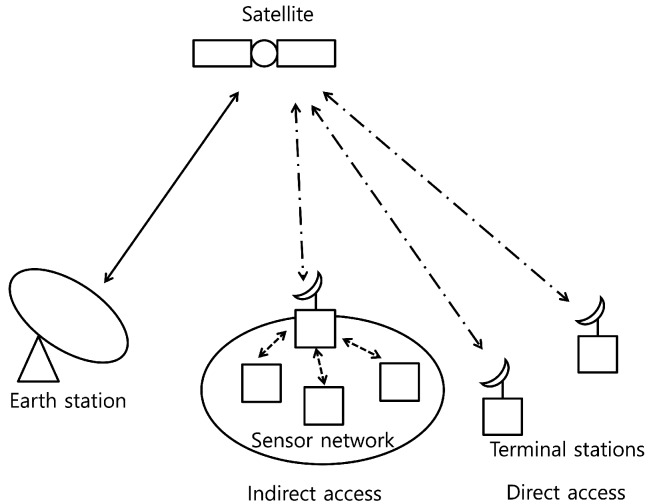
Satellite communication networks supporting Internet of Things (IoT).

As an emerging wireless network technology, wireless sensor networks, especially Internet of Things (IoT) networks, have gathered a lot of attention as a solution to various applications, such as an environmental monitoring and data collection for a smart community like smart city [1,2]. Because there are needs for data transmission from isolated and wide areas in IoT networks, the wide cell coverage of the IoT is a key requirement. Therefore, the integrated networks of the existing wireless sensor network and satellite communications is an essential component for achieving the goal of the various IoT applications [3,4,5]. As shown in Figure 1, two different satellite links can be considered: Direct and indirect access links. To gather the information in the remote areas, e.g., ocean and desert, a sink node (terminal station) collects the information from sensors and forwards this collected information to a monitoring station (Earth station) via a satellite using an indirect access link. Therefore, the satellite link is considered as a backbone link for the wireless sensor networks. On the other hand, a terminal station can be a sensor node and it transmits its own data to the Earth station via a satellite.

Because the wide network coverage, e.g., global coverage, of wireless sensor networks, such as IoT, is one of their most significant requirements, satellites have become an important part of wireless communication systems. Moreover, a satellite is not a dedicated resource but is rather shared by service providers with their own services because it is one of the most expensive network entities. The assigned bandwidth for a given satellite communication service is called a transponder. Transponder bandwidth is typically fixed in the satellite during the design process and is not finely controllable after the satellite is launched. Each transponder provides a connection with dedicated bandwidth and transmission power between two points, i.e., Earth and terminal stations. With such a fixed transponder, there are major disadvantages in terms of bandwidth efficiency and transmit power control. For example, if a satellite customer needs a slightly wider bandwidth than a provided transponder bandwidth, the customer should purchase one more transponder, which is a waste of bandwidth. To solve such a problem, the bandwidth of the subchannel in the digital channelizer is minimized for fine granularity of the transponder. In general, for efficient implementation, a digital filter bank is used for a digital channelizer [6,7,8].

Because of their wide transmission coverage, satellite communication systems are more vulnerable to interference that is generated in a wide area [9,10,11]. Interference is a threat for satellite communication systems and services because they are used for various significant purposes including military wireless communication networks. There are many different sources of interference: It can be generated by malicious users or generated unintentionally by nonlinear analog components in transceivers. Self-interference, which is an echo version of the transmitted signal, can be generated in a full-duplex operation [12]. The capability of interference mitigation in a satellite is an important issue because without it, the quality of service cannot be guaranteed and a satellite can be useless in an environment with interference. To mitigate this interference problem, cognitive interference detection and cancellation techniques are required [9,13,14,15].

Various approaches are adopted for interference cancellation, depending on the properties of the target signal. As cognitive radios have attracted much attention from researchers to improve spectrum utilization, various detection methods to sense primary signals in cognitive radios have been proposed [16,17,18]. If the signal waveform is unknown, an energy detection method, which is one of the simplest detection schemes, is generally used to detect the signal [19,20,21].

One of the typical types of interference is a continuous wave (CW) tone interference which can be simply generated by a malicious user to interrupt satellite communications. In other cases, CW tone interference can be produced by inter-modulation of the internal clock and carrier frequencies used in a satellite transceiver. In this work, we focus on CW tone interference detection and cancellation. A digital channelizer based on a digital filter bank is used for interference cancellation by nulling the subchannel corrupted by the interference, before inputting to the synthesis filter of the filter bank. In this interference cancellation scheme, it is shown that a tone interference whose power is lower than the power of the desired signal component in a single subchannel does not need to be cancelled out because the cancellation causes more distortion of the desired signal than the interference. To detect a subchannel interfered by a CW tone signal, we employ an energy detection which measures the received signal power per subchannel and compares it with a threshold. With this simple approach, we can achieve the reasonable detection performance, i.e., low false alarm and high detection probabilities. However, this requires a long time, i.e., many samples of the signal, for accurate detection. To reduce the detection time, an improved method is also proposed. In the proposed method, we exploit the structure of a fast Fourier transform (FFT) operation which is the simplest filter bank. To verify the performance of the proposed method, we analyze the false alarm and detection probabilities of the interference detection algorithm and perform computer simulations under various conditions.

### 1.1. Contributions

The main contributions of this paper are summarized as follows:

- Cognitive interference detection and cancellation schemes are proposed. The proposed approaches can be distinguished from the existing interference cancellation methods because they are based on a digital channelizer which is an essential part in a satellite, needed to utilize transponders bandwidth-efficiently.
- In the proposed interference cancellation, a range of the interference power which should be cancelled out is evaluated. For too weak interference, the interference cancellation causes more severe signal distortion than the interference level and the cancellation is not needed.
- The detection and false-alarm probabilities of the proposed interference detection schemes are analyzed. The validity of the analysis is also verified through simulations. Based on the analytical results, we can provide a guideline in designing the cognitive interference cancellation system under various conditions.

### 1.2. Notations

For a random variable *x*, $x∼N(\mu ,\sigma^{2})$ means that *x* is Gaussian distributed with mean μ and variance $\sigma^{2}$. E[x] denotes an expectation of the random variable. $Q\left(x\right)\left(=\frac{1}{\sqrt{2\pi }}\int_{x}^{\infty }e^{-t^{2}/2}dt\right)$ represents the tail probability of a Gaussian distribution.

## 2. System Model

**Figure 2:**
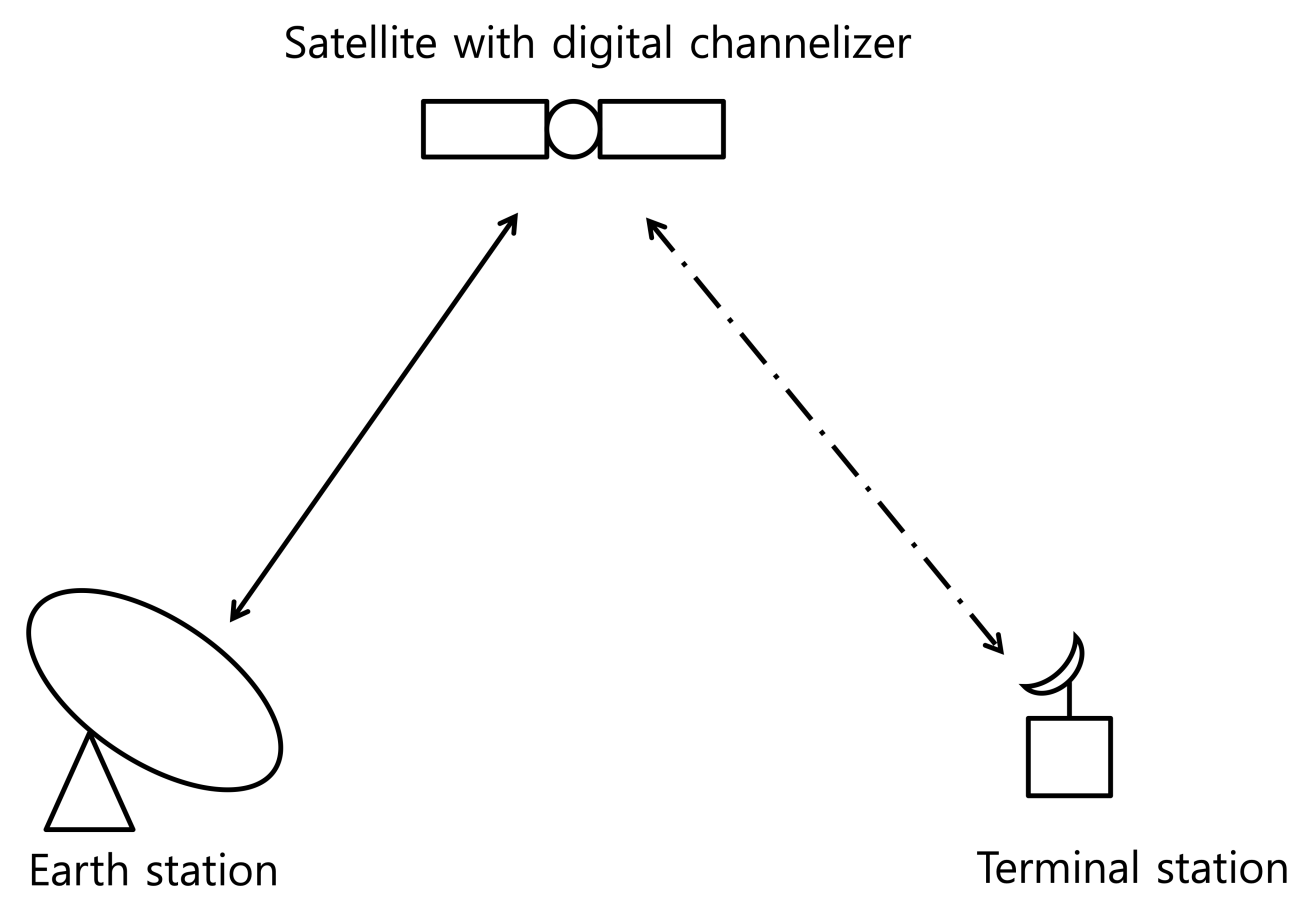
Satellite communication channel with Earth and terminal stations, and satellite with a digital channelizer.

**Figure 3:**
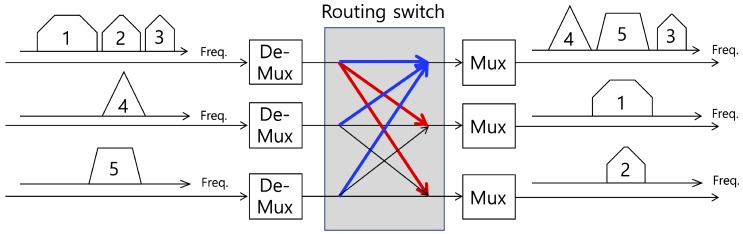
Functional block diagram and operational example of digital channelizer.

In this study, a point-to-point satellite communication channel with an Earth station as well as a satellite and terminal station is considered as shown in Figure 2. In typical satellite communication, a satellite is not dedicated to a single link (or service), i.e., several links (or services) share a satellite for their own purposes. The total bandwidth of the satellite consists of several transponders, which are clusters including radio receivers, frequency translators, and transmitters. A satellite customer purchases a transponder, i.e., a dedicated block of bandwidth on a satellite for a period of time and provides a satellite communication service. To utilize the transponder, i.e., bandwidth, efficiently, a satellite employs a digital channelizer with many subchannels. With the digital channelizer, the transponder bandwidth and power can be finely controlled. For example, a carrier frequency of the transponder can be changed and the transmit power is also controlled using a switching and multiplexing functions of the channelizer. Figure 3 shows an operational example and overall functional diagram of a digital channelizer.

In this work, we focus on a cognitive interference cancellation at a satellite by using a digital channelizer with *N* subchannels. Details on the digital channelizer and its implementation will be discussed in the next section. The signal received by the satellite as an input of the digital channelizer is given by

(1) y(Ni+k)=x(Ni+k)+s(Ni+k)+w(Ni+k),i=0,1,⋯,k=0,⋯,N−1,

where x(·), s(·) and w(·) are the desired wideband signal, CW tone interference, and additive white Gaussian noise (AWGN), respectively. Because we consider a 1024-subchannel channelizer, we make a block with 1024 samples. In Equation (1), *i* is a block index and *k* is the time domain sample index in the block. With the channelizer, the time-domain signal block, [y(Ni),y(Ni+1),⋯,y(Ni+N−1)], is transformed to the frequency-domain signal block, $\left[\bar{y}\left(Ni\right),\bar{y}\left(Ni+1\right),\cdots ,\bar{y}\left(Ni+N-1\right)\right]$. Therefore, the output of the digital channelizer is expressed by

(2) $$\begin{array}{cc} \bar{y}\left(i,0\right) & =\bar{y}\left(Ni\right)=\bar{x}\left(i,0\right)+\bar{w}\left(i,0\right)\\ \bar{y}\left(i,1\right) & =\bar{y}\left(Ni+1\right)=\bar{x}\left(i,1\right)+\bar{w}\left(i,1\right)\\ \vdots & \;\;\;\;\;\;\;\;\;\;\vdots \;\;\;\;\;\;\;\;\;\;\vdots \\ \bar{y}\left(i,n\right) & =\bar{y}\left(Ni+n\right)=\bar{x}\left(i,n\right)+\bar{s}\left(i,n\right)+\bar{w}\left(i,n\right)\\ \vdots & \;\;\;\;\;\;\;\;\;\;\vdots \;\;\;\;\;\;\;\;\;\;\vdots \\ \bar{y}\left(i,N-1\right) & =\bar{y}\left(Ni+N-1\right)=\bar{x}\left(i,N-1\right)+\bar{w}\left(i,N-1\right). \end{array}$$

In Equation (2), it is assumed that the frequency of the interference s(·) is in the *n*-th subchannel. In the cognitive interference cancellation, the subchannel with the tone interference is identified and removed. After the cancellation, the satellite combines the uncorrupted subchannels and transmits (relays) the wideband signal to the receiver on Earth. As needed, switching and multiplexing functions, as well as power control are performed before transmission at the satellite.

## 3. Digital Channelizer

In satellite communication, a satellite operates as a relay between Earth and terminal stations. In general, satellites have multiple purposes, e.g., satellite broadcasting services and satellite communication services, and can change the carrier frequency, bandwidth and gain of the received signal form a transmitter (e.g., an Earth station) before relaying it to the receiver (e.g., a terminal station). A digital channelizer is used for efficient implementation of switching and multiplexing functionality. In this section, a digital channelizer based on a digital filter bank is briefly reviewed.

**Figure 4:**
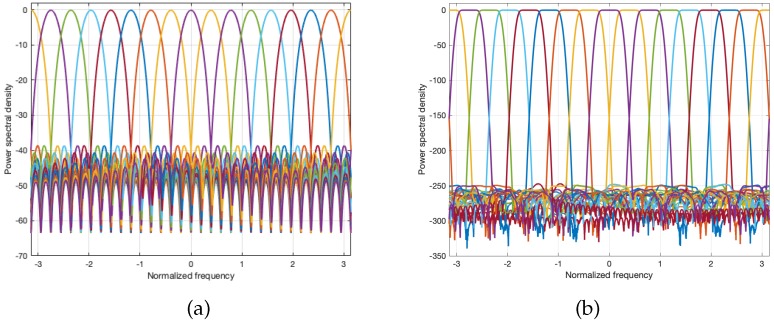
Power spectral density of filter banks with 16 subchannels: (**a**) Discrete Fourier transform (DFT) filter bank and (**b**) near-perfect reconstruction (NPR) polyphase filter bank.

The main function of a digital channelizer is to divide the received signal into multiple subchannels and to reconstruct the wideband signal by combining these multiple subchannels. The filter that divides the wideband signal into multiple subchannels with uniform bandwidth is called an analysis filter bank and that which combines the multiple subchannels is called a synthesis filter bank. Discrete Fourier transform (DFT) is one of the simplest filter banks, i.e., digital channelizer, and it is efficiently realized using an FFT algorithm. However, the stopband attenuation and flatness of the passband of the DFT filter bank are not sufficient for switching and multiplexing functions in a digital channelizer. The FFT operation is regarded as a digital channelizer with a rectangular finite impulse response (FIR) filter. Therefore, the spectrum mask of each subchannel becomes a sinc function as shown in Figure 4a. By designing a sophisticated lowpass FIR (prototype) filter instead of a rectangular FIR (prototype) filter, both the stopband attenuation and passband flatness of the subchannel in the digital channelizer can be improved. Therefore, the objective function of the digital channelizer design is given by the weighted sum of two measures: The stopband attenuation of a lowpass (prototype) filter, and sum of the squared magnitude responses of all subchannels. For example, a quadrature mirror filter (QMF) banks design method was proposed by Johnston [22]. Jian [23] and Nguyen [24] also proposed other filter design methods.

In this study, a near-perfect reconstruction (NPR) polyphase filter bank is used in the design of a digital channelizer with 1024 subchannels. The frequency response of the designed filter bank with 16 subchannels is shown in Figure 4b. Because the frequency response of a 1024-subchannel filter bank is not visible, we show that of 16-subchannel filter bank which is designed with the same method. As shown in the figure, the designed digital channelizer with an NPR polyphase filter bank has negligible error in the sum of magnitude response and significant stopband attenuation. Therefore, is can be concluded that the designed filter bank can be used as a digital channelizer for satellite communications.

## 4. Cognitive Interference Cancellation

The CW tone interference is one of the most popular interferences in wireless communication. CW tone interference can be generated by two different sources: Malicious transmitters (especially, a jammer), and inter-modulation and total harmonic distortion of nonlinear elements in an RF/analog transceiver. In this work, we focus on the single tone interference detection and cancellation in a satellite with a digital channelizer.

Tone interference detection includes both the decision on the existence, and frequency estimation of the CW tone interference (e.g., finding a subchannel corrupted by the interference). For cognitive interference cancellation of the CW tone interference, however, the exact estimation of frequency, amplitude and phase is crucial. For example, inaccurate estimation of the interference frequency can crease another interference source. To avoid the complex process of interference estimation and cancellation, interference cancellation based on a digital channelizer is proposed. In detail, we divide the received wideband signal into a large number of subchannels, i.e., 1024 subchannels, and identify the subchannel with CW tone interference and remove it. As expected, as the number of subchannels increases, the interference cancellation performance gain increases as well as this requires higher implementation complexity from a digital channelizer. Hence, a reasonable number of subcarriers can be determined based on the maximum operating signal-to-noise ratio (SNR) of a satellite link. For example, if the maximum operating SNR is given by about 20 dB, an interference signal with power −30 dB less than the desired wideband signal is ignored, i.e., the performance degradation caused by this interference is negligible. As one of the typical satellite communication standards, digital video broadcasting-second generation (DVB-S2) requires 16.05 dB SNR for 32 amplitude phase shift keying (APSK) modulation and 9/10 low density parity check (LDPC) coding rate in ideal AWGN channels [25]. Assuming a 4 dB implementation margin considering the practical impairments such as carrier frequency offset, timing offset, phase noise, nonlinear power amplifier and so on, the maximum operating SNR is higher than 20 dB, and −30 dB interference less than the desired signal is ignored because such low interference does not degrade the system performance.

When the interference power is −30 dB lower than the desired signal, we do not need to detect and cancel the interference. Additionally, when the interference is falsely detected and one of subchannels are nullified, the distortion of the wideband signal should be less than –30 dB. Therefore, we set the number of subchannels to 1024, to cancel the interference of which power is –30dB less than the desired signal power. Based on this requirement, we can determine the number of subchannels in a digital channelizer as 1024. In the following subsections, we introduce the two interference detection methods based on the digital channelizer.

### 4.1. Simple Interference Detection

The cognitive interference detection and cancellation scheme is developed with a 1024-subchannel digital channelizer. For the *m*-th subchannel, an average signal power is measured as

(3) $$T\left(m\right)=\frac{1}{L}\sum_{i=1}^{L}{\left|\bar{y}\left(i,m\right)\right|}^{2},$$

where *L* is the number of samples for power measurement for each subchannel. In this work, this is denoted by the number of subchannel blocks used for power measurement. The output of the digital channelizer for the the *n*-th subchannel, which is assumed to include the tone interference, is given by

(4) $$\begin{array}{c} \left\{\begin{array}{cc} \bar{y}\left(i,n\right)=\bar{s}\left(i,n\right)+\bar{x}\left(i,n\right)+\bar{w}\left(i,n\right), & \mathrm{under}\;H_{1},\\ \bar{y}\left(i,n\right)=\bar{x}\left(i,n\right)+\bar{w}\left(i,n\right), & \mathrm{under}\;H_{0}, \end{array}\right. \end{array}$$

where $H_{0}$ and $H_{1}$ denote the null and alternative hypothesis, i.e., under $H_{0}$ where we assume the tone interference does not exist, and under $H_{1}$ where the tone interference is assumed to exist. Other subchannels without interference are given by

(5) $$\bar{y}\left(i,m\right)=\bar{x}\left(i,m\right)+\bar{w}\left(i,m\right),\;\;\;\mathrm{for}\;m\neq n.$$

Detection of tone interference is done by comparing the measured average signal power with a given threshold λ. To correctly detect the existence of the interference, the following conditions are satisfied:

(6) $$\begin{array}{c} \left\{\begin{array}{cc} T(n)\geq \lambda ,\\ T(m)<\lambda , & m\neq n. \end{array}\right. \end{array}$$

For no false alarm, i.e., the correct detection of absence of the interference, the following condition is used:

(7) $$\begin{array}{cc} T(m)<\lambda , & \;\;\mathrm{for}\;\mathrm{all}\;m\in \{0,\cdots ,1023\}. \end{array}$$

The proposed cognitive interference detection scheme is easy to implement because the structure of the digital channelizer is inherently utilized. The required additional complexity is negligible. However, the detection performance of the simple algorithm is a problem. With a given number of blocks *L*, e.g, L≤10 (total number of time domain samples ≤10240), the detection probability for a given false alarm rate is too low to cancel the interference. In other words, a large number of signal blocks is needed, i.e., the output of the digital channelizer, to achieve the acceptable detection performance.

### 4.2. Improved Interference Detection

**Figure 5:**
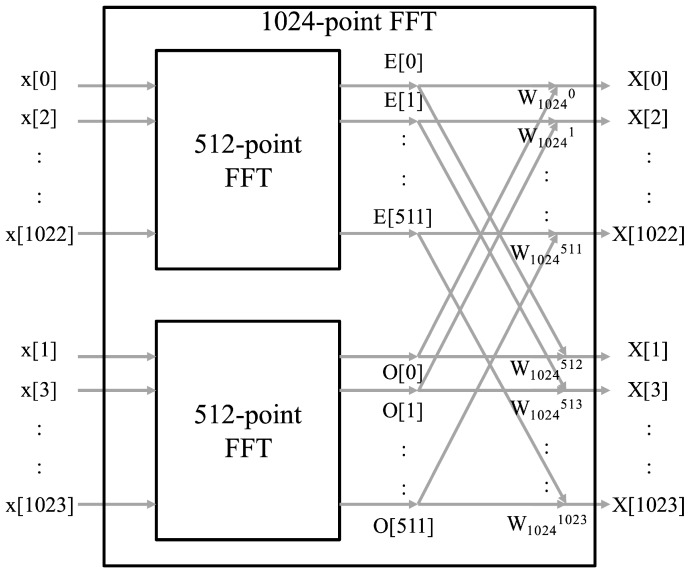
Structure of fast Fourier transform (FFT) using radix-2 algorithm. (x[k] is time-domain input with 1024 samples, X[k] is the 1024-point FFT output, E[k] and O[k] are the 512-point FFT outputs with even- and odd-indexed samples, respectively, and $W_{1024}^{k}=\exp\left(\frac{-i2\pi k}{1024}\right)$.)

**Figure 6:**
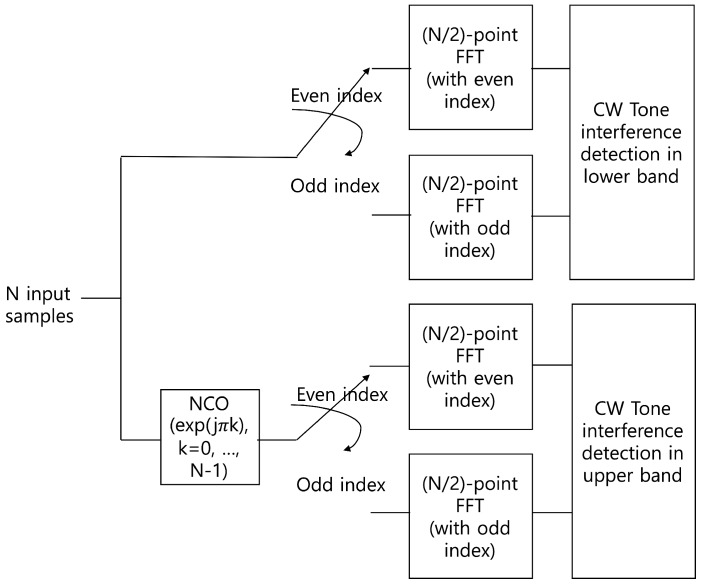
Overall structure of the proposed interference detector where N=1024.

**Figure 7:**
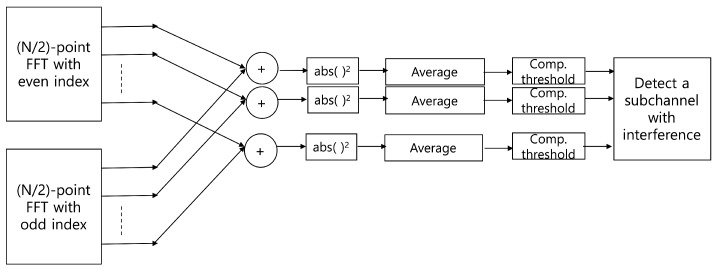
Detailed structure of the continuous wave (CW) tone interference detection block in Figure 6 where N=1024.

To improve the interference detection performance, the previous method is modified using an FFT operation. Because the FFT is one of the simplest filter banks, we can use it as a simple digital channelizer even though it has poor stopband attenuation and passband flatness. These characteristics of the FFT channelizer are not a significant problem in detecting CW tone interference. On the other hand, the low complexity and flexibility of FFT is an advantage in interference detection. In the process of the 1024-point FFT with a radix-2 algorithm, we can automatically obtain two 512-point FFT outputs with even- and odd-indexed input of 1024 input samples in the time domain. In Figure 5, the structure of the 1024-point FFT including two 512-point FFT with even- and odd-indexed input samples is shown. Therefore, the output of the 512-point FFT represents the spectrum of the lower half band of the total bandwidth which consists of the 1024-point FFT input signal. Because the input of the 512-point FFT consists of every second sample, i.e., even- and odd-indexed samples, the bandwidth of each subchannel for the 512-point FFT is identical to that of the 1024-point FFT. However, the number of subchannels for the 512-point FFT is half of that of the 1024-point FFT. To obtain the spectrum of the upper half band, i.e., subchannels of the upper half band, a frequency up-shift of the 1024 input samples is adopted by using a numerical oscillator, exp(jπk) for k=0,1,⋯. The overall structure of the proposed interference detector is illustrated in Figure 6. The detailed block of CW interference detection in the lower and upper bands is shown in Figure 7. As shown in the figure, two output samples from the subchannels in the lower and upper bands are accumulated and averaged for *L* subchannel blocks. The square of its magnitude is compared with the predefined threshold to determine the existence of interference in each subchannel. Subsequently, the decision statistic for the improved interference detection is given by

(8) $$T_{im}\left(m\right)=\frac{1}{L}\sum_{i=1}^{L}\left[|{\bar{y}}_{e}\left(i,m\right)+{\bar{y}}_{o}{\left(i,m\right)|}^{2}\right],$$

where ${\bar{y}}_{e}\left(i,m\right)$ and ${\bar{y}}_{o}\left(i,m\right)$ denote the *m*-th FFT output sample (*m*-th subchannel) in the *i*-th block of the even- and odd-indexed input signals. For the lower band, the range of *m* is given by m=0,⋯,255,768,⋯,1023. For the upper band, m=256,⋯,767.

To detect the subchannel corrupted by interference, the decision statistics for the subchannels are compared with the threshold. The condition for the interference detection existing in the *n*-th subchannel is given by

(9) $$\begin{array}{c} \left\{\begin{array}{cc} T_{im}\left(n\right)\geq \lambda ,\\ T_{im}\left(m\right)<\lambda , & m\neq n. \end{array}\right. \end{array}$$

Similar to (7), the condition for the no false alarm event is given by

(10) $$T_{im}\left(m\right)<\lambda ,\;\mathrm{for}\;\mathrm{all}\;m\in \left\{0,\cdots ,1023\right\}.$$

The improved method provides two 512-subchannel outputs but the bandwidth of all 512 subchannels is half of the total signal bandwidth. Because the range of the frequency detection in the improved method is just half of the total bandwidth, the frequency ambiguity problem cannot be avoided. This means that tone interference in the higher band is also detected in the lower band interference detection process and vice versa. For example, if the *n*-th subchannel in the higher band (n≥512) is assumed to be corrupted with interference, the interference is also detected in the (n−512)-th subchannel in the lower band. However, this frequency ambiguity is simply overcome with the output of the 1024-point FFT. In the above example, the actual interfered subchannel can be identified by comparing the *n*-th and (n−512)-th subchannels in the 1024-point FFT output. As mentioned previously, two 512-point FFTs with even- and odd-indexed samples are by-products of the 1024-point FFT. Therefore, the complexity of the additional process required to solve the frequency ambiguity is much smaller than that of the other processes in the interference detection.

Note that the FFT is used to detect a subchannel with interference, and channelization is performed by an NPR polyphase filter bank for enhancement of both the stopband attenuation and passband flatness of the subchannels.

## 5. Performance Analysis

In this section, the performance of the proposed cognitive interference detection algorithm is analyzed. First, we derive the detection and false alarm probabilities of the simple method with 1024 subchannels. To this end, we obtain the distribution of the decision statistics for both hypotheses $H_{0}$ and $H_{1}$. The decision statistic in each subchannel is given by Equation (3) and its distribution for both hypotheses is expressed by

(11) $$\begin{array}{cc} T(n) & ∼\left\{\begin{array}{cc} N\left(\sigma_{s}^{2}+\sigma_{w}^{2},\frac{2\sigma_{w}^{4}}{L}\right), & \mathrm{under}H_{1},\\ N\left(\sigma_{w}^{2},\frac{2\sigma_{w}^{4}}{L}\right), & \mathrm{under}H_{0}, \end{array}\right. \end{array}$$

(12) $$\begin{array}{cc} T(m) & ∼N\left(\sigma_{w}^{2},\frac{2\sigma_{w}^{4}}{L}\right),\;\;\mathrm{for}\;m\neq n, \end{array}$$

where $\sigma_{s}^{2}$ denotes the CW tone interference power and $\sigma_{w}^{2}$ is the variance of $\bar{x}\left(\cdot ,\cdot \right)+\bar{w}\left(\cdot ,\cdot \right)$, i.e., the sum power of the desired signal and AWGN components in each subchannel. It is also assumed that the desired signal in the frequency domain, $\bar{x}\left(\cdot ,\cdot \right)$, follows Gaussian distribution by the central limit theorem because it is the weighted sum of *N* modulated symbols in the time domain [26,27].

Based on the above distribution, we can derive the probability to detect the interference correctly with a threshold λ in each subchannel as follows:

(13) $$\begin{array}{cc} p_{d} & =Pr\left\{T\left(\cdot \right)\geq \lambda |H_{1}\right\}\\ \; & =Q\left(\frac{\lambda -\left(1+\gamma \right)\sigma_{w}^{2})}{\sigma_{w}^{2}\sqrt{2/L}}\right), \end{array}$$

where $\gamma =\frac{\sigma_{s}^{2}}{\sigma_{w}^{2}}$ represents SNR with respect to the CW tone interference [19,20]. Similarly, the false alarm probability is given by

(14) $$\begin{array}{cc} p_{fa} & =Pr\left(T\left(\cdot \right)<\lambda |H_{0}\right)\\ \; & =Q\left(\frac{\lambda -\sigma_{w}^{2}}{\sigma_{w}^{2}/\sqrt{2/L}}\right). \end{array}$$

With the detection and false alarm probabilities for a single subchannel, we can evaluate the overall performance of the interference detection considering all subchannels.

(15) $$\begin{array}{cc} P_{d} & =Pr\left(T\left(n\right)\geq \lambda |H_{1}\right)Pr\left(T\left(m\right)<\lambda |H_{0},\;\mathrm{for}\;\mathrm{all}\;m\neq n\right)\\ \; & =p_{d}{\left(1-p_{fa}\right)}^{1023} \end{array}$$

(16) $$\begin{array}{cc} P_{fa} & =Pr\left(T\left(m\right)>\lambda |H_{0},\;\mathrm{for}\;\mathrm{any}\;m=0,\cdots ,1023\right)\\ \; & =1-Pr\left(T\left(m\right)<\lambda |H_{0},\;\mathrm{for}\;\mathrm{all}\;m=0,\cdots ,1023\right)\\ \; & =1-{\left(1-p_{fa}\right)}^{1024}. \end{array}$$

For the improved approach, the decision statistics are distributed as follows:

(17) $$\begin{array}{cc} T_{im}\left(n\right) & ∼\left\{\begin{array}{cc} N\left(4\sigma_{s}^{2}+2\sigma_{w}^{2},\frac{8\sigma_{w}^{4}}{L}\right), & \mathrm{under}H_{1},\\ N\left(2\sigma_{w}^{2},\frac{8\sigma_{w}^{4}}{L}\right), & \mathrm{under}H_{0}, \end{array}\right. \end{array}$$

(18) $$\begin{array}{cc} T_{im}\left(m\right) & ∼N\left(2\sigma_{w}^{2},\frac{8\sigma_{w}^{4}}{L}\right),\;\;\mathrm{for}\;m\neq n, \end{array}$$

because two 512-point FFT outputs with even- and odd-indexed inputs are added before measuring the signal power. Therefore, the detection and false alarm probabilities for each subchannel are given by

(19) $$\begin{array}{cc} p_{d}^{\prime } & =Q\left(\frac{\lambda^{\prime }-2\left(1+2\gamma \right)\sigma_{w}^{2})}{2\sigma_{w}^{2}\sqrt{1/L}}\right), \end{array}$$

(20) $$\begin{array}{cc} {p_{fa}}^{\prime } & =Q\left(\frac{\lambda^{\prime }-2\sigma_{w}^{2}}{2\sigma_{w}^{2}\sqrt{2/L}}\right), \end{array}$$

where $\lambda^{\prime }$ is a predefined threshold in the improved method. By considering the lower or upper band, the overall detection and false alarm probabilities of interference detection are expressed by

(21) $$\begin{array}{cc} P_{d}^{\prime } & =p_{d}^{\prime }{\left(1-p_{fa}^{\prime }\right)}^{511}, \end{array}$$

(22) $$\begin{array}{cc} P_{fa}^{\prime } & =1-{\left(1-p_{fa}^{\prime }\right)}^{512}. \end{array}$$

## 6. Numerical Results

For the evaluation of the cognitive interference detection and cancellation performance, and verification of analytical results, we conduct computer simulations under various conditions.

**Figure 8:**
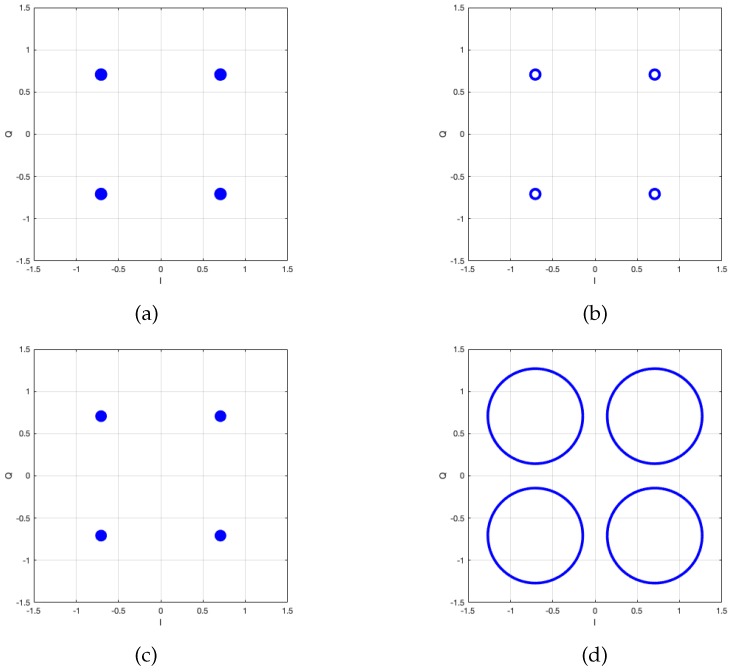
Quadrature phase shift keying (QPSK) constellation with and without interference cancellation depending on the interference power: (**a**) With interference cancellation in –25 dB lower interference than the desired signal, (**b**) without interference cancellation in –25 dB lower interference than the desired signal, (**c**) with interference cancellation in –5 dB lower interference than the desired signal, and (**d**) without interference cancellation in –5 dB lower interference than the desired signal.

First, the minimum interference power that should be cancelled out is evaluated. As mentioned previously, interference with a power lower than a certain level does not need to be cancelled because its cancellation causes higher degradation of the desired wideband signal than the interference. For example, Figure 8 shows constellation of the desired wideband signal with and without interference cancellation when the CW tone interference power is –25 dB and –5 dB lower than the desired wideband signal. The wideband signal is modulated by quadrature phase shift keying (QPSK) and uses single carrier transmission. The number of subchannels is set to be 1024 and a single tone interference with a normalized frequency of $\frac{200}{1024}\pi$ radians/sample is added. The frequency of the interference is not changed in this study. In a subchannel with interference, the power of AWGN is much lower than the power of the interference and desired signal components, i.e., $\left[\right|\bar{s}{\left(\cdot ,\cdot \right)|}^{2}]\gg E[|\bar{w}\left(\cdot ,\cdot \right){|}^{2}]$ and $E[|\bar{x}{\left(\cdot ,\cdot \right)|}^{2}]\gg E[|\bar{w}\left(\cdot ,\cdot \right){|}^{2}]$ in Equation (2). Therefore, we neglect the AWGN term, i.e., $\bar{w}\left(\cdot ,\cdot \right)=0$. Additionally, we normalize the desired signal in the whole band. Therefore, the power of the desired signal component in each subchannel becomes 1/1024, i.e., –30 dB.

Assuming that the subchannel index corrupted by interference is perfectly known, Figure 8a–c show the constellation with interference cancellation with different interference power. The proposed method cancels the interference by omitting the corrupted subchannel. This means that one of the 1024 components of the desired signal is also omitted in the cancellation process. Even though the interference is correctly cancelled out, we cannot obtain the ideal QPSK constellation as shown in Figure 8a–c. The error vector magnitude (EVM), i.e., the inverse of the effective SNR, is –30 dB because 1/1024 of the desired signal along with the CW tone interference is cancelled out. The definition of EVM is the ratio of the mean square of the error vector to the power of the reference constellation, i.e., ideal constellation. That is, the EVM in dB scale is as follows:

(23) $$\begin{array}{c} EVM\left(dB\right)=10\log_{10}\left(\frac{E[|C_{mea}-C_{ref}{|}^{2}]}{E[|C_{ref}{|}^{2}]}\right), \end{array}$$

where $C_{mea}$ and $C_{ref}$ denote the measured and reference constellations, respectively. The numerator and denominator in Equation(23) stand for the error caused by noise and interference in signal constellations and the power of the ideal signal, respectively. Therefore, the EVM is regarded as the inverse of the signal-to-noise (-plus-interference) ratio. On the other hand, Figure 8b and Figure 8d show the constellation of the desired signal without interference cancellation under low and high interference conditions. The EVM of the constellation in Figure 8c is –25 dB because the CW tone interference power is –25 dB lower than the desired wideband signal. Figure 8d shows the constellation without cancellation of the higher interference, i.e., –5 dB lower than the desired wideband signal, and the EVM of this case is –5 dB.

**Figure 9:**
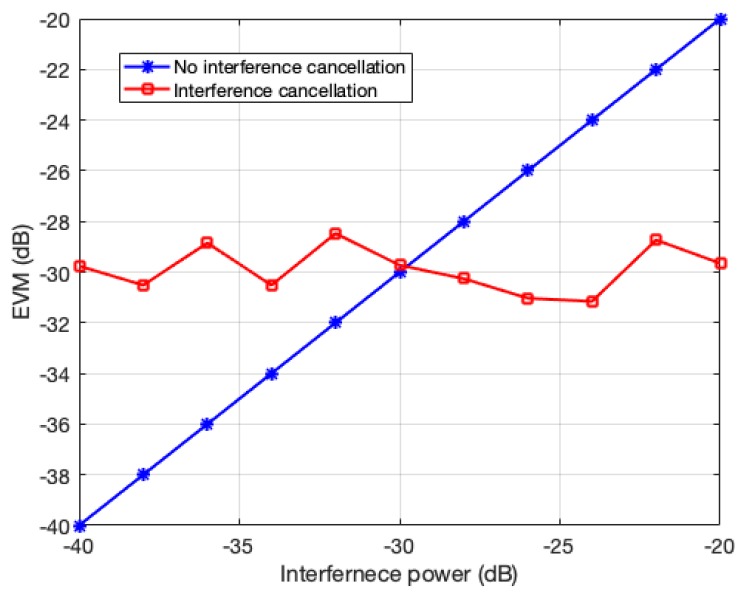
Error vector magnitude (EVM) performance with and without interference cancellation depending on interference power.

Figure 9 shows the EVM results with and without interference cancellation when the CW tone interference power varies from –40 to –20 dB (lower than the desired signal power in the whole bandwidth). In this simulation, we use the same condition as in Figure 8. The desired signal is modulated with a single carrier QPSK, 1024 subchannels are used, and a single tone interference at $\frac{200}{1024}\pi$ radians/sample frequency is added. When the interference is cancelled, the EVM of the desired signal is about –30 dB regardless of the interference power. However, if the interference is not cancelled out, the EVM increases with the interference power because the error is caused by the interference.

**Figure 10:**
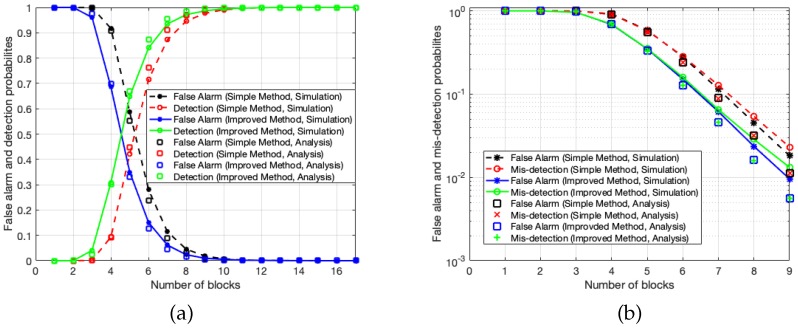
Interference detection performance of simple and improved approaches with respect to the number of blocks, *L*, when interference power is –23 dB lower than the desired signal, λ=3 for the simple method and $\lambda^{\prime }=6$ for the improved method: (**a**) False alarm and detection probabilities and (**b**) false alarm and misdetection probabilities.

To evaluate the interference detection performance of the proposed simple and improved methods, we show the false alarm and detection (or misdetection) probabilities in Figure 10a,b. Here, we set the power of the CW tone interference as –23 dB lower than the desired wideband signal and its frequency is $\frac{200}{1024}\pi$ radians/sample. The total number of subchannels is 1024 as in the previous simulations. This means that the CW tone interference has 7 dB higher power than the desired signal in the subchannel with interference. The detection thresholds for the simple and improved approaches are set at λ=3 and $\lambda^{\prime }=6$, respectively. In Figure 10a, false alarm and detection probabilities are shown as the number of subchannel blocks, *L*, increases. The simulation results (lines with markers), in addition to the analytical performance (only markers) for both the simple and improved methods are shown. As expected, the false alarm probability decreases and detection probability increases with the number of blocks, *L*. When L≥10, we can achieve nearly perfect detection performance. The improved method requires a smaller number of subchannel blocks than the simple one in terms of both false alarm and detection probabilities. Roughly, one subchannel block can be saved with the improved method, with a cost of implementation complexity (or detection range of interference frequency). Additionally, the analytical results are well matched with the simulation ones. To investigate a more accurate performance of the interference detection, we plot the false alarm and misdetection probabilities in a log scale in Figure 10b.

## 7. Conclusions

In satellite communication, a digital channelizer can be adopted in a satellite to efficiently support multiple transponders. In this scenario, a digital channelizer based on a filter bank was used for cognitive interference cancellation with low complexity. This is done by detecting the subchannel with tone interference and omitting this subchannel in the frequency domain, i.e., after an analysis filter, the interference can simply get eliminated. Even though this simple approach is effective for cancelling tone interference, better interference detection performance was achieved by an improved method exploiting the properties of an FFT operation. By making two channelizer outputs with even- and odd-indexed inputs, we achieved an enhanced detection performance with the same number of subchannel blocks. With simulations and theoretical analysis, the performance of both the simple and improved approaches for interference detection were verified. It was also shown that the cancellation of interference with power less than that of the desired signal component in one subchannel is not needed. The proposed cognitive interference cancellation schemes exploit the inherent characteristics of the digital channelizer required for the flexible utilization of transponders. With the analytical and numerical results, the effectiveness of the proposed methods is verified and the design guideline of the cognitive interference cancellation based on the digital channelizer is provided.
